# Environmental DNA detection tracks established seasonal occurrence of blacktip sharks (*Carcharhinus limbatus*) in a semi-enclosed subtropical bay

**DOI:** 10.1038/s41598-020-68843-0

**Published:** 2020-07-16

**Authors:** Bautisse D. Postaire, Judith Bakker, Jayne Gardiner, Tonya R. Wiley, Demian D. Chapman

**Affiliations:** 1 Department of Biological Sciences, Florida International University, 3000 NE 151st Street, North Miami, FL 33181 USA; 2 Division of Natural Sciences, New College of Florida, 5800 Bayshore Rd, Sarasota, FL 34243 USA; 3 Havenworth Coastal Conservation, 5120 Beacon Road, Palmetto, FL 34221 USA

**Keywords:** Animal migration, Conservation biology, Molecular ecology

## Abstract

The integration of eDNA analysis into the population assessment and monitoring of sharks could greatly improve temporal and spatial data used for management purposes. This study aimed to compare eDNA detection against well-established seasonal changes in blacktip shark (*Carcharhinus limbatus*) abundance in Terra Ceia Bay (FL, USA). We used a species-specific real-time PCR approach to detect *C. limbatus* eDNA in the bay on a near monthly basis from spring through mid-fall in 2018 and 2019. Previous studies have shown that *C. limbatus* give birth in the bay in early summer and immature sharks occur there until late fall, when decreasing water temperatures cause them to move offshore and southwards. Water samples (2 L) were collected (4–6 per month) and filtered in the field, with each then being subjected to real-time PCR. *Carcharhinus limbatus* ‘positive’ filters were significantly more commonly collected during the April-July sampling period than during the August-October sampling period. While following the predicted pattern, eDNA concentration was generally too low for accurate quantification. Our results show that *C. limbatus* eDNA detection follows known seasonal residency patterns consistently over 2 years of monitoring. Species-specific eDNA analysis using real-time PCR could therefore represent a cost-effective, scalable sampling tool to facilitate improved shark population monitoring in semi-enclosed marine habitats.

## Introduction

Many shark populations have been heavily impacted by overexploitation and environmental disturbances^1–6^. Effective assessment, monitoring and management of these predators will rely on accurate knowledge of species spatiotemporal distribution and abundance trends, which are often incomplete or absent^7^. Commonly employed methods to obtain these data, such as sampling with gillnets or longlines, are invasive and resource intensive, which often leads to a strong dependence on non-standard data from fisheries^8–10^. Cost-effective and scalable alternatives are needed to improve our understanding of shark distributions and population dynamics.

The application of environmental DNA (eDNA) analysis has emerged as a new approach to detect macroorganisms from trace DNA in water samples^11–13^. eDNA approaches are based on the isolation, amplification and sequencing of DNA traces from skin cells, egestion, and metabolic waste left behind in the environment^14,15^. They offer a promising avenue for non-invasive, cost-effective, and scalable monitoring studies of aquatic organisms that can achieve very high replication independent of fisheries^16,17^. Shark eDNA represents a naturally rare target for sampling given their positions as upper level predators (naturally lower densities compared to species occupying lower trophic levels), coupled with low abundances due to overexploitation in many areas^18^, suggesting a high probability of negative results^17,19^. Nevertheless, eDNA analysis has recently been applied for the detection of sharks and their relatives and has proven to have potential for presence/absence and biodiversity studies^10,20–27^. As has been shown for other marine organisms, there is also growing evidence indicating that the quantity of eDNA may be related not only to species presence, but also to abundance^16,21,28–32^. Although promising as a method for shark detection, more research in well-studied ecosystems is necessary to understand whether eDNA monitoring can also be used to track population dynamics over time.

Here we set out to test whether species-specific eDNA analysis could detect a well-established seasonal change in blacktip shark (*Carcharhinus limbatus*) occurrence in Terra Ceia Bay (FL, USA). We developed and tested a species-specific real-time PCR assay targeting a short stretch of the *C. limbatus* mitochondrial NADH2 gene to be amplified from filtered water samples collected in this subtropical semi-enclosed estuary in the spring, summer and fall months in 2018–2019. Terra Ceia Bay is ideally suited for this purpose because *C. limbatus* abundance peaks in spring and early summer and troughs in late summer, fall, and winter as a result of parturition, early mortality and migration out of the bay^1,33^. Correspondingly, we predicted that *C. limbatus* eDNA detection, measured in this study by the proportion of positive filters (i.e. *C. limbatus* detected) and the relative DNA concentrations measured on these filters, would be significantly higher in spring and early summer than in late summer and fall.

## Material and methods

### Study species and area

**Figure 1:**
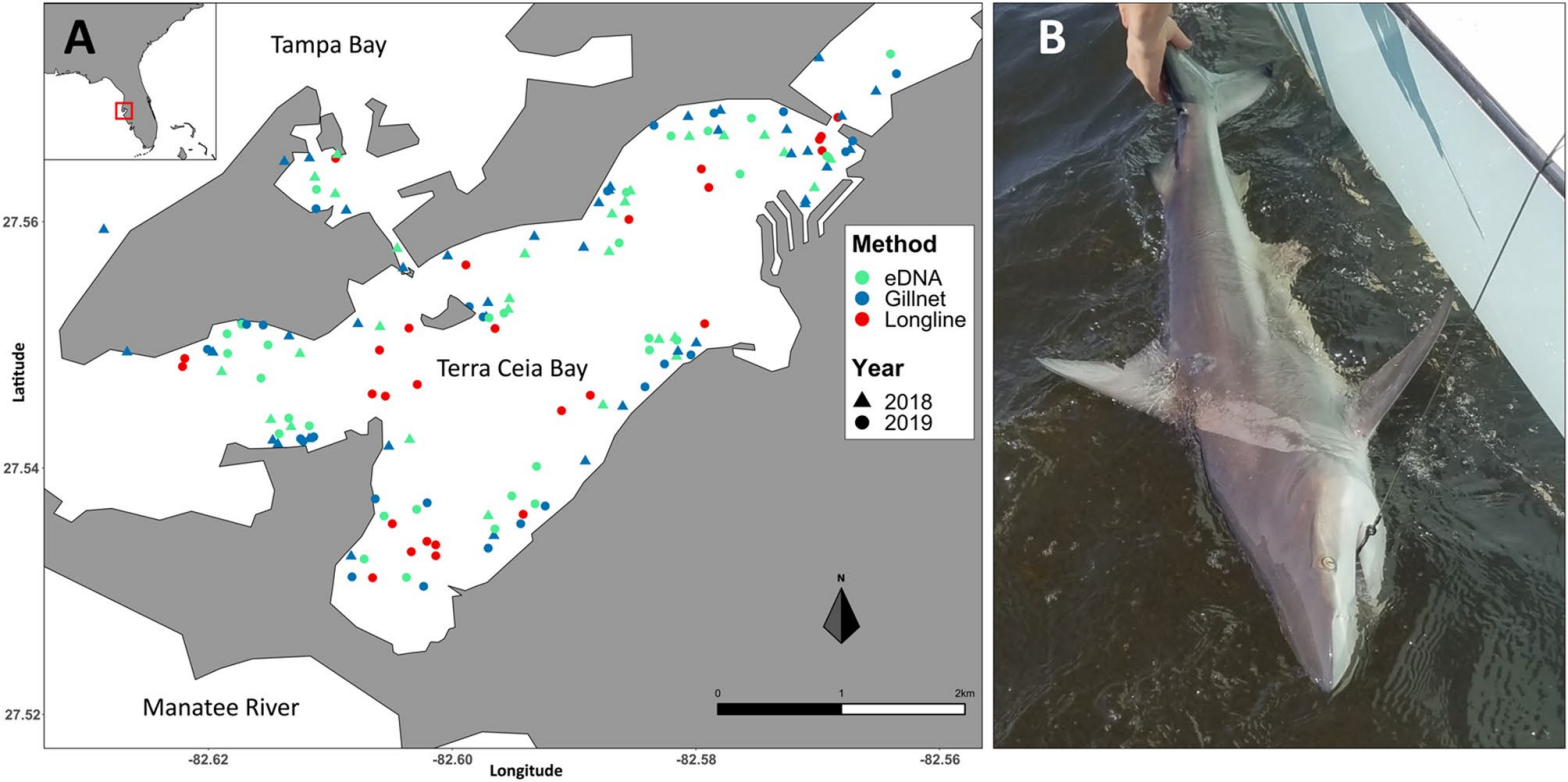
(**A**) Map of sampling locations in Terra Ceia Bay (FL, USA). Indicated are in green the eDNA sampling sites, in blue the gillnet sites in 2018 and 2019, and in red circles the longline sampling sites in 2019. Sampling events are aggregated per year and method (i.e. not all sites were sampled every month, see Supplementary Material 1 for detail). (**B**) A juvenile *Carcharhinus limbatus*, caught, tagged and released in Terra Ceia Bay (photo credit: T. R. Wiley). Map was generated with R v 3.4.0 (https://www.R-project.org/) using polygons extracted from *Google Earth*, (https://earth.google.com/web/).

The blacktip shark (*Carcharhinus limbatus*; Fig. 1B) is a cosmopolitan species, encountered globally in tropical and subtropical waters^34–36^. It is a commercially and recreationally important species in the southeast U.S. and Gulf of Mexico shark fisheries, as well as being one of the top four species in the global shark fin trade^37,38^. The species has moderately slow growth rates and low fecundity; producing pups in alternating years after a 10–12 month gestation period, giving birth to 4–7 pups^34,39^. This leads to low natural recruitment abilities, rendering their populations susceptible to stock collapses from overexploitation. However, the U.S. *C. limbatus* population is well managed and sustainably fished. In this study we refer to four life-stage categories: neonate, young of the year, immature, and mature, following the definitions of Castro^35^. Neonates are animals with an open umbilical scar, these scars close within the first 2–3 weeks of life. Young of the year (YOY) have closed umbilical scars, which are typically still visible. YOY can also be distinguished from older immature animals based on size^40^. Mature males are distinguished from immature males based on claspers that are elongated and calcified, while for females, 50% size-at-maturity was used to distinguish between immature and mature animals^39^. Along the Florida Gulf Coast, neonate, YOY, and immature *C. limbatus* seasonally use inshore nursery areas including Terra Ceia Bay^1,41,42^. Terra Ceia Bay is a small (~ 5 km × 1.5 km), shallow (~ 4 m maximum depth) bay with a narrow (~ 1 km) opening into Tampa Bay (Fig. 1A). As *C. limbatus* is a migratory species that prefers temperatures above 21 °C, there is an influx of immature sharks into the bay during April after an absence for most of the winter. Gravid females enter the bay for parturition in May and June^1,33,34^. Natural mortality of neonates is highest within the first 15 weeks of life, which is reflected in a sharp decrease in their abundance from July into August. The remaining young of the year and immature animals leave the bay and migrate southward during the fall months^1,33^. These patterns have held with continued sampling in the bay until the present, although some immature animals now stay in the bay until later in the year and return earlier, possibly because waters have warmed in recent years (Gardiner, unpublished data).

### Shark sampling

Shark surveys in Terra Ceia Bay were conducted as part of the GulfSPAN survey (www.fisheries.noaa.gov/southeast/endangered-species-conservation/shark-and-sawfish-surveys-and-tagging), a fishery-independent effort to assess shark diversity and relative abundance along the Florida Gulf Coast in order to inform stock assessments. All protocols for the handling and use of animals were approved by the Institutional Animal Care and Use Committee at the University of South Florida (protocol # W IS00004541) and sampling was conducted in accordance with Florida Fish and Wildlife Conservation Commission Special Activities Licenses SAL-18-1666-SRP and SAL-19-1666-SRP. The surveys are conducted each year from April to October. In order to conserve limited resources for this survey, sampling is not conducted from November to March when shark abundances are known to be very low due to decreased water temperatures during these winter months. The primary sampling method of this project for 2018 was monofilament gillnet. However, as this method primarily captures neonate and YOY sharks, it was considered not to be an appropriate comparison on its own to eDNA, which is shed by all life stages. Therefore, bottom longline gear was also deployed on a monthly basis in 2019 to sample a wider variety of life stages and to provide a better comparison to eDNA when combined with gillnets. For each month of sampling in 2019, catches were summed over 3–4 longline and gillnet stations and used to calculate monthly catch-per-unit-effort (CPUE), defined as the number of individuals caught per station per hour. All sampling coordinates and details of the fishing gear are summarized in Supplementary Material 1 and Supplementary Material 2, respectively.

### eDNA collection and processing

Water samples were collected on a monthly basis concurrent with GulfSPAN surveys during June–October 2018 and April–October (except July) 2019. Four to six water samples were collected at each sampling location per monthly survey, immediately after gear is set in order to avoid contamination from captured animals. eDNA samples were collected in mid-water to avoid catching suspended sediment, ensuring that only recently released eDNA was collected^43,44^. A total of 58 water samples of 2 L each (Supplementary Material 1) were collected with a Kemmerer type water sampler and vacuum filtration was carried out immediately after collection with a Pegasus Alexis peristaltic pump (www.fondriest.com). The cups containing the hydrophilic polyethersulfone (PES) filters (Pall Corporation; 47 mm diameter; 0.45 µm pore size) were placed in a clean, iced cooler (containing only eDNA samples) for transport to the laboratory. The filters containing sample filtrates were stored in sterile 5.0 mL cryogenic screw-cap vials containing silica beads. The silica beads function as a desiccator, drying out the filters, preventing the DNA from degradation^26^. The filters were then stored at − 20 °C until extraction. DNA extraction from half of each filter was performed using the DNeasy PowerSoil Kit (www.qiagen.com), following the manufacturers’ protocol, with three additional specifications. As the outer circumference of the filter does not contain any DNA (due to placement of the filter cup), it was cut off and discarded. The remainder of the half filter was cut into small pieces prior to being added to the bead tubes for the first step of the extraction process. The vortexing (bead-beating) step of the protocol was performed in a shaker at 58 °C (for 10 min) instead of at room temperature, in order to maximize the DNA yield. Genomic DNA was eluted into 100 μL and frozen at − 20 °C until further processing.

### Real-time PCR assay development

#### Primer design

Primers were designed to target a 149 bp fragment of the mitochondrial (mtDNA) nicotine adenine dinucleotide dehydrogenase subunit 2 (NADH2) gene within the *C. limbatus* genome, while excluding cross amplification with other elasmobranch species known to co-occur in the study area (*Rhizoprionodon terraenovae, R. porosus, Carcharhinus brevipinna, C. acronotus, C. leucas, Sphyrna mokarran, S. lewini, S. zygaena*, and *S. tiburo*). Primers were designed manually using Geneious Prime, version 2019.2.3 (https://www.geneious.com). NADH2 sequences from *C. limbatus* and the non-target shark species were downloaded from GenBank (Supplementary Material 3) and aligned using the Muscle 3.8.425 plugin in Geneious^45^. Forward (588 F-limbatus-NADH2: 5′-TGCCCCCAATCTCACCTTAC-3′) and reverse (776 R-limbatus-NADH2: 5′-CCGGAAAGTGGGGGTAATCC-3′) primers were designed to amplify the fragment of interest exclusively in *C. limbatus*. This was accomplished by maximizing the number of mismatches in the ligation sites of both primers in the nine exclusion species. In order to confirm primer specificity, they were first tested by applying conventional PCR to total genomic DNA (gDNA) extracted from eight *C. limbatus* individuals and from eight individuals per each of the nine species known to co-occur in the study area. Genomic DNA was extracted from tissue samples using the DNeasy Blood & Tissue kit (www.qiagen.com) following the manufacturer’s protocol. Each amplification reaction was performed in a total volume of 15 µL and consisted of: 7.5 µL of Master Mix ‘Applied 2×’ (Applied Biosystems), 3.5 µL of DNase/RNase-free water (Fisher Scientific), 1 µL of each primer (10 µM) and 2 µL of genomic DNA (10 ng/µL). The PCR profile included an initial denaturing step of 95 °C for 2 min, 35 cycles of 95 °C for 30 s, 63 °C for 30 s and 72 °C for 30 s and a final extensions step of 72 °C for 5 min. The quality of all amplifications was assessed by electrophoresis, running the products through a 1.5% agarose gel stained with Gel Red (Gentaur, Kampenhout, Belgium) and visualized on a UV light platform. Subsequently, the primers were tested on the real-time PCR platform for amplification of tissue-derived gDNA from *C. limbatus* and the non-target species. PCR products were sequenced in both directions to confirm species identity, using an ABI 3730 genetic analyzer (Applied Biosystems). Sequences were checked using Geneious.

#### Real-time PCR

Concentrations of *C. limbatus* NADH2 fragments in our eDNA samples were checked by running six replicate reactions for each sample, using the Chai Open PCR System platform (Chai Biotechnologies, Santa Clara, CA, USA). The real-time PCR recipe was optimized to a final volume of 15 µL, containing 7.5 µL of PowerUp SYBR Green Master Mix (Applied Biosystems), 2.5 µL of DNase/RNase-free water (Fisher Scientific), 1 µL of each primer (5 µM) and 3 µL of eDNA template. The real-time PCR program consisted of 50 °C for 2 min, an initial denaturation step at 95 °C for 2 min, followed by 80 cycles of 95 °C for 30 s, 62 °C for 30 s, and 72 °C for 30 s. A final melting curve analysis step at 60 °C for 1 min was followed by an increasing ramp temperature of 5 °C per second to reach 95 °C for 5 min. Using 80 amplification cycles in real-time PCR is not standard protocol when working with tissue extractions, but with appropriate negative controls it has been found to be useful for detecting the presence of eDNA that which is usually present in very low concentrations^16^. The melt curve protocol was used to detect primer dimers, to check specificity by determining that a single amplicon was produced, and to obtain a melting temperature (Tm) value for that sample, which may then be compared with the standard curve of *C. limbatus* DNA. Each real-time PCR run consisted of 16 reactions (two 8-well PCR strips per run), including two positive (13.1 × 10^−1^ ng/μL gDNA), and two negative (DNase/RNase-free water) controls. Cq values of the standard concentrations were compared between independent runs to check for consistency and replicability of results.

#### Primer efficiency, LOD and LOQ

Primer efficiency, limit of detection (LOD), and limit of quantification (LOQ) were assessed following the protocol and curve fitting method described in Klymus et al.^46^. For the standard curve, tenfold dilutions (in ddH_2_O) of *C. limbatus* gDNA derived from tissue extractions were used. The dilution series spanned seven orders of magnitude, ranging from 13.1 × 10^−1^ to 13.1 × 10^−7^ ng/μL, measured with a Qubit 2.0 fluorometer (Invitrogen, Carlsbad, CA, USA). Each dilution was run ten times and reported Cq (cycle quantification) values were used to determine primer efficiency, LOD, and LOQ^46^. LOD and LOQ were determined at a concentration of 13.1 × 10^−6^ ng/μL of gDNA (the conc. at which at least 95% of the replicates amplified with a CV of 54% or less, see “Results” section). Amplification efficiency was determined by plotting Cq values against gDNA dilutions and calculating the linear slope and the coefficient of determination (R^2^) value.

### Contamination control

Strict adherence to contamination control procedures was followed during all field and laboratory stages. In the field, disposable gloves were used and changed between each sample. A section of the boat was allocated as a clean area for water collection and filtration. Work surfaces, water collection and filtration equipment were cleaned prior to and after each use with a 50% bleach solution. To prevent cross-contamination between samples, filtration was performed using disposable, single-use filter funnels, which were subsequently stored individually in two layers of double sealed plastic bags prior to being stored on ice. All eDNA sample processing and analyses were conducted in the Crustacean Genomics and Systematics Lab at Florida International University where there had never been any shark tissue or PCR product present. Extractions, pre-PCR preparations and (post)PCR procedures were physically and temporally separated. All laboratory equipment was cleaned with bleach solution and subsequently exposed to UV sterilization (including ddH_2_O used for PCR reactions) for 15 min, before and after each use. Pertaining to filter extractions, bags containing the individual filters were cleaned on the outside with bleach prior to entering the lab. All work surfaces were cleaned with 50% bleach solution prior to and after each use, and between processing of individual samples. All equipment used for the extractions was likewise cleaned with bleach between each individual sample. Disposable gloves were used and changed between each sample or in addition, more often when deemed appropriate. Aerosol barrier pipette tips were used for all laboratory procedures. *C. limbatus* tissue extraction was performed months prior to the onset of the eDNA laboratory work, in a separate dedicated tissue extraction room. Separate laboratory rooms, located in different parts of the building, were used for PCR and real-time PCR experiments. No equipment was shared between the laboratories and commuting between the different laboratories within the same day was prohibited, this included colleagues who were not part of this study. In order to identify potential contamination, negative controls were added at multiple stages. Negative controls were collected in the field by filtering 2 L of tap water, processed as standard samples. In the laboratory, DNA extraction blanks (elution buffer from extraction kit) and PCR blanks were included.

### Data analysis

Cq and melting temperature (Tm) values were used to determine the presence/absence of *C. limbatus* eDNA in a sample, by comparison with those of the standard curves and the positive controls. The Cq value indicates the cycle at which the fluorescence produced by the DNA amplification in a sample surpasses a preset threshold of fluorescence background noise, which is a proxy for the quantity of eDNA present in a sample. When a sample has a Cq value, it means that target DNA is present assuming there is no non-specific amplification. The Cq value represents the PCR cycle at which amplification is first detected and is positively correlated with the template DNA present in the sample. Filters were considered positive when at least two out of the six replicates produced a Cq value, in combination with an associated Tm value close (± 2 °C) to the mean Tm of the standard curve (78.2 °C). For each filter sample, the average Cq of all positive replicates were calculated to obtain a single value per filter.

Because many detections presented Cq values below LOQ and LOD (Cq>42.09, see “Results” section), they were treated as qualitative data (presence or absence of target DNA on the filter)^46^. A Chi square proportion test was performed to test for a difference in the proportion of *C. limbatus* positive filters between the high (April–July) and low abundance (August–October) seasons over 2018 and 2019, and the catch data (CPUE) was also compared between the same two seasons in 2018 and 2019 using the Wilcoxon signed-rank test. In order to determine the correlation between the proportion of positive filters and catch data (CPUE) per month, a non-parametric Spearman correlation was performed. All statistical analyses were performed in R v 3.4.0 (https://www.R-project.org/).

## Results

### Assay efficiency and sensitivity

The custom primers amplified only the NADH2 target sequence in *C. limbatus* and not in any of the non-target species tested, confirming primer specificity of the assay for both conventional and real-time PCR. Additionally, the identity of real-time PCR amplified eDNA was confirmed to be *C. limbatus* by Sanger sequencing 14 of the eDNA real-time PCR products (using the amplifying primers). These *C. limbatus* sequences have been deposited in GenBank (www.ncbi.nlm.nih.gov/genbank/), accession numbers are recorded in Supplementary Material 4. The standard curve produced from the tenfold serial dilution of the *C. limbatus* gDNA extraction showed a linear relationship between the Cq value and the log of the starting gDNA concentration (R^2^ > 0.99). The average slope was − 3.872, corresponding to a PCR efficiency ~ 81.24%. The limit of detection (LOD) and limit of quantification (LOQ) were calculated following a curve-fitting model^46^. LOD and LOQ were determined at a gDNA concentration of 13.1 × 10^−6^ ng/μL (the conc. at which at least 95% of the replicates amplified with a CV of 54% or less); the average Cq was 42.09.

### Carcharhinus limbatus eDNA detection in Terra Ceia Bay

*C. limbatus* eDNA was detected in water samples collected in Terra Ceia Bay. A total of 27 of the 58 water samples (46.6%) were considered positive for this species based on our criteria (Supplementary Material 4). All negative controls produced negative results. A significantly higher number of filters were positive during the spring/early summer high abundance season compared to the late summer/early fall low abundance season (X^2^ = 9.6107, df = 1, p value = 0.001934, alternative hypothesis: two-sided).

**Figure 2:**
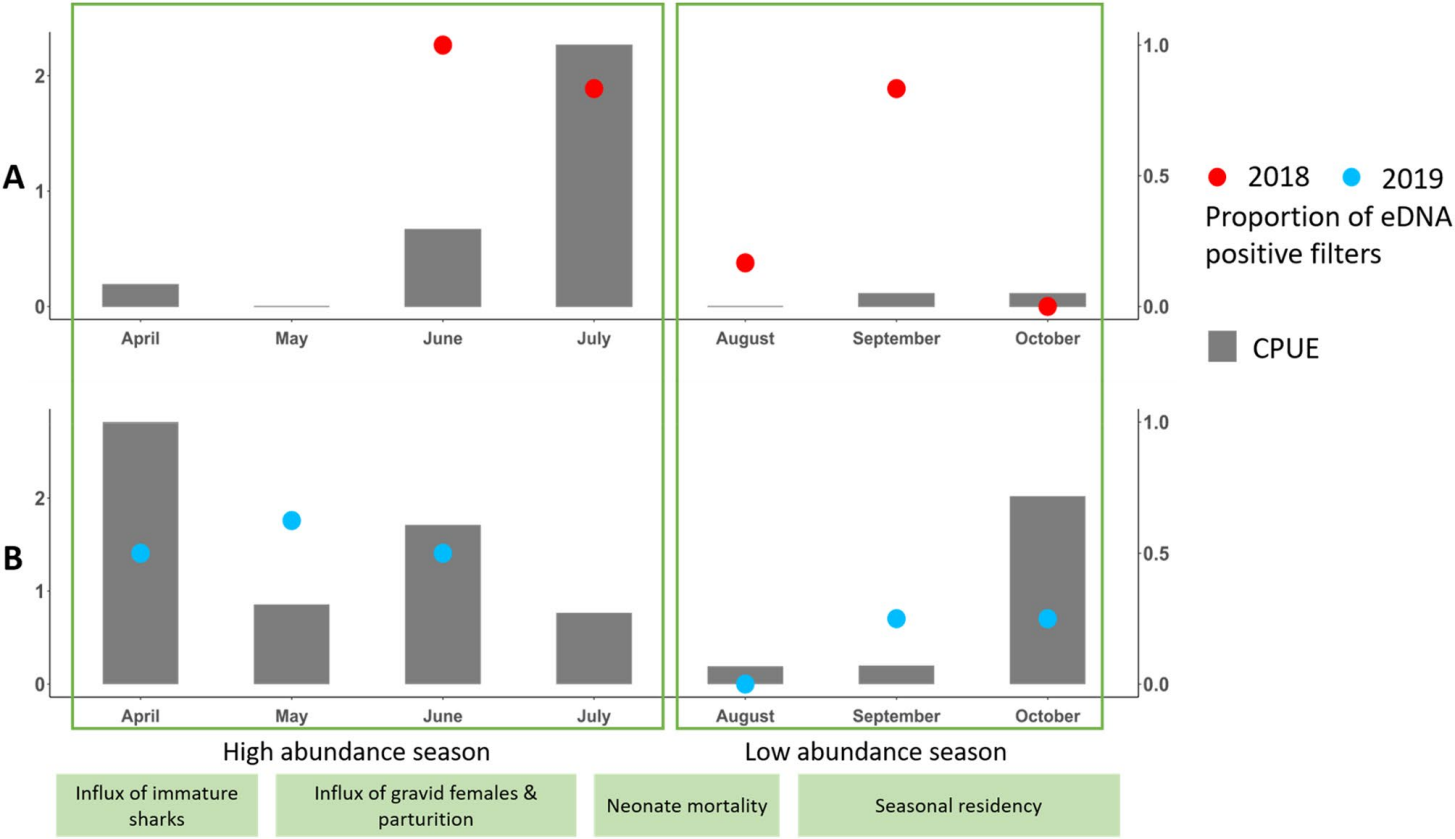
Gillnet, longline and eDNA results for 2018 and 2019. Panel (**A**) shows the *C. limbatus* gillnet catch data (left y-axis) and the proportion of filters yielding *C. limbatus* DNA in 2018 (right y-axis). Panel (**B**) shows the proportion of filters yielding *C. limbatus* DNA in 2019 (right y-axis) and combined gillnets and longline catches in 2019 (left y-axis). Values for fishing are expressed as CPUE (individuals per station/h).

A total of 24 *C. limbatus* individuals were caught by 40 gillnets in 2018. A total of 37 individuals were captured across 27 longlines and 3 by 27 gillnets in Terra Ceia Bay from April to October 2019. Catch data per shark can be found in Supplementary Material 5. CPUE was not significantly different between the defined sampling periods, although near zero CPUE was recorded in August and September co-incident with low eDNA detection (Fig. 2). No correlation was detected between monthly CPUE and proportion of positive eDNA detections (rho = 0.4735797, p value = 0.08716) over the sampling period.

## Discussion

The application of eDNA analysis specifically for sharks and their relatives has recently gained momentum, and studies largely focus on providing proof-of-concept that their eDNA can be detected^10,20–23^. In this study we developed a real-time PCR assay that detected *C. limbatus* eDNA in water samples from Terra Ceia Bay. A subset of the eDNA PCR products were directly sequenced and proved to be from the target species and locus. The assay was able to detect target DNA down to concentrations as low as 13.1 pg/μL. However, in order for eDNA methods to advance for conservation management purposes beyond this application, it is imperative to test whether it can also be a reliable indicator of changes in species presence over time, which may be related to abundance on various temporal and spatial scales^16^. This is not only important for the detection of population declines or changes in geographic distribution, but also for the determination of seasonal residency patterns, as we trialed here. We found that *C. limbatus* positive filters were collected significantly more often in spring to mid-summer than in late summer and fall. This change is coincident with changes in local abundance due to parturition, mortality and seasonal migration of *C. limbatus* in Terra Ceia Bay, based on data previously collected through extensive fishing surveys and acoustic telemetry studies^1,33,41^. While there may be concerns relating to eDNA transport and the interpretation of results in a (semi-enclosed) marine system, many studies have shown that there is only minimal eDNA transport by water movement on both horizontal^13,47–50^ and vertical^51^ spatial scales. This indicates that the seasonal pattern detected in eDNA detection resulted from temporal changes in the abundance of the species in the bay.

We found that traditional real-time PCR analysis for the detection of eDNA from naturally rare vertebrate target organisms had some limitations. While the qualitative results (i.e. positive/negative filters) were robust and followed the expected pattern of *C. limbatus* abundance, most measurements were below LOD and LOQ of the assay. Even though the quantitative results also followed the expected pattern of *C. limbatus* abundance, they could thus not be reliably reported. While detections of target eDNA with values below LOD are expected due to the rarity of the target species, and are thus still considered true positives^46,52,53^, quantifying eDNA concentrations accurately provides even more information relevant for management. Given the rarity of shark eDNA targets and the limitations of real-time PCR we suggest that future applications of this and similar assays should use digital droplet PCR (ddPCR)^54^ for the high resolution quantification of rare target eDNA. This has recently been trialed for the detection of elasmobranch eDNA^20,23,55^, but has yet to be implemented for the spatio-temporal tracking of abundance.

Our study suggests that species-specific real-time PCR eDNA analysis may provide useful information for shark conservation and management, but it is important to assess its potential weaknesses compared to traditional methods. First and foremost, eDNA methods do not provide information on size, condition, developmental stage and sex, or allow for tagging or sampling of the target animals, which makes it difficult to interpret changes in detection over time without a-priori information. It could also be misleading if eDNA shedding rates vary with size, sex, season, or physiological state. Although complicated due to the requirements and restrictions related to using live sharks for controlled experiments^20^, more fundamental studies investigating (the factors related to) shark eDNA shedding, accumulation, persistence and degradation could greatly contribute to our understanding and interpretation of eDNA results relating to spatiotemporal abundance patterns.

Overall, we found that species-specific real-time PCR eDNA analysis offered several advantages compared to the fishing surveys conducted simultaneously. Catch rates of longlines and gillnets in the bay were highly variable and zero-inflated, which made statistical analysis challenging. The number of positive eDNA filters was significantly different between the seasonal sampling periods, while the CPUE data were not. Closer inspection of the CPUE data revealed that this failure to detect a seasonal difference was partly due to the one anomalous high catch (8 sharks) on one longline in October 2019, otherwise catch rates were near zero in August and September similar to the eDNA results. In addition, the time needed to collect and filter each water sample was trivial (minutes) compared to setting, soaking, and checking and hauling longlines and gillnets (hours) and could be achieved with two people, as opposed to a minimum of four. Field time and labor are significant cost barriers to the replication of shark surveys; eDNA replication would be substantially less expensive. After the assay was developed and validated, laboratory analysis of filters took only days and the reagent cost per filter (including extraction and 6 PCR replicates) was ~ USD$11. Using ddPCR instead of real-time PCR would increase the cost per sample but there may be scenarios where real-time PCR is sufficient on its own or as a first check to detect positive or negative filters. Pertaining to our project in particular, eDNA sampling could provide data coverage over the winter months, while keeping the costs much lower than when fishing surveys would take place year-round. Extended eDNA sampling throughout the winter could potentially reveal spatio-temporal changes due to rising water temperatures. eDNA sampling can verify until when some animals stay in the bay, and how much earlier they return. And perhaps, whether some of the immature animals do not leave the bay anymore at all during the winter.

This study represents an important step in the development of eDNA methods for the detection and monitoring of elasmobranchs. We developed a species-specific real-time PCR eDNA assay and found that *C. limbatus* detection in our semi-enclosed study area fluctuated seasonally according to *a-priori* expectations for two consecutive sampling years. While eDNA methods have inherent limitations because individuals are not physically caught and counted, and we still lack basic information on the process of eDNA shedding in sharks, this approach is relatively inexpensive and scalable while also being non-invasive. We therefore conclude that eDNA sampling should be considered for integration into existing standardized shark monitoring programs that use traditional methods to track population dynamics over time. eDNA analysis has the potential to become a powerful complementary monitoring tool for shark population assessment and management, offering a potential for replication that is difficult to match using other fishery-independent methods.

## Supplementary information

Supplementary Information 1.

Supplementary Information 2.

Supplementary Information 3.

Supplementary Information 4.

Supplementary Information 5.

## References

[1] Heupel MR, Simpfendorfer CA (2002). Estimation of mortality of juvenile blacktip sharks, *Carcharhinus limbatus*, within a nursery area using telemetry data. Can. J. Fish. Aquat. Sci..

[2] Robbins WD, Hisano M, Connolly SR, Choat JH (2006). Ongoing collapse of coral-reef shark populations. Curr. Biol..

[3] Camhi MD, Valenti SV, Fordham SV, Fowler SL, Gibson C (2009). The Conservation Status of Pelagic Sharks and Rays.

[4] Ward-Paige CA (2010). Large-scale absence of sharks on reefs in the greater-Caribbean: A footprint of human pressures. PLoS One.

[5] Worm B (2013). Global catches, exploitation rates, and rebuilding options for sharks. Mar. Policy.

[6] Spaet JLY, Berumen ML (2015). Fish market surveys indicate unsustainable elasmobranch fisheries in the Saudi Arabian Red Sea. Fish. Res..

[7] Dulvy NK (2014). Extinction risk and conservation of the world’s sharks and rays. Elife.

[8] Wheeler QD (2004). Taxonomy: Impediment or expedient?. Science.

[9] Lodge DM (2012). Conservation in a cup of water: Estimating biodiversity and population abundance from environmental DNA. Mol. Ecol..

[10] Simpfendorfer CA (2016). Environmental DNA detects critically endangered largetooth sawfish in the wild. Endanger. Species Res..

[11] Baker CS, Steel D, Nieukirk S, Klinck H (2018). Environmental DNA (eDNA) from the wake of the whales: Droplet digital PCR for detection and species identification. Front. Mar. Sci..

[12] Miya M (2015). MiFish, a set of universal PCR primers for metabarcoding environmental DNA from fishes: Detection of more than 230 subtropical marine species. R. Soc. Open Sci..

[13] Port JA (2016). Assessing vertebrate biodiversity in a kelp forest ecosystem using environmental DNA. Mol. Ecol..

[14] Ji Y (2013). Reliable, verifiable and efficient monitoring of biodiversity via metabarcoding. Ecol. Lett..

[15] Taberlet P, Coissac E, Hajibabaei M, Rieseberg LH (2012). Environmental DNA. Mol. Ecol..

[16] Lacoursiere-Roussel A, Rosabal M, Bernatchez L (2016). Estimating fish abundance and biomass from eDNA concentrations: Variability among capture methods and environmental conditions. Mol. Ecol. Resour..

[17] Jerde CL (2019). Can we manage fisheries with the inherent uncertainty from eDNA?. J. Fish Biol..

[18] Ward-Paige CA (2010). Large-scale absence of sharks on reefs in the greater-Caribbean: A footprint of human pressures. PLoS One.

[19] Furlan EM, Gleeson D, Hardy CM, Duncan RP (2016). A framework for estimating the sensitivity of eDNA surveys. Mol. Ecol. Resour..

[20] Schweiss KE, Lehman RN, Drymon JM, Phillips NM (2019). Development of highly sensitive environmental DNA methods for the detection of Bull Sharks, *Carcharhinus leucas* (Müller and Henle, 1839), using Droplet Digital^TM^ PCR. Environ. DNA.

[21] Weltz K (2017). Application of environmental DNA to detect an endangered marine skate species in the wild. PLoS One.

[22] Gargan LM (2017). Development of a sensitive detection method to survey pelagic biodiversity using eDNA and quantitative PCR: A case study of devil ray at seamounts. Mar. Biol..

[23] Lafferty KD, Benesh KC, Mahon AR, Jerde CL, Lowe CG (2018). Detecting Southern California’s white sharks with environmental DNA. Front. Mar. Sci..

[24] Sigsgaard EE (2016). Population characteristics of a large whale shark aggregation inferred from seawater environmental DNA. Nat. Publ. Gr..

[25] Truelove NK, Andruszkiewicz EA, Block BA (2019). A rapid environmental DNA method for detecting white sharks in the open ocean. Methods Ecol. Evol..

[26] Bakker J (2017). Environmental DNA reveals tropical shark diversity and abundance in contrasting levels of anthropogenic impact. Sci. Rep..

[27] Boussarie G (2018). Environmental DNA illuminates the dark diversity of sharks. Sci. Adv..

[28] Buxton AS (2017). Seasonal variation in environmental DNA in relation to population size and environmental factors. Sci. Rep..

[29] Doi H (2017). Environmental DNA analysis for estimating the abundance and biomass of stream fish. Freshw. Biol..

[30] Yamamoto S (2016). Environmental DNA as a ‘snapshot’ of fish distribution: A case study of Japanese jack mackerel in Maizuru Bay, Sea of Japan. PLoS One.

[31] Levi T (2019). Environmental DNA for the enumeration and management of Pacific salmon. Mol. Ecol. Resour..

[32] Salter I, Joensen M, Kristiansen R, Steingrund P, Vestergaard P (2019). Environmental DNA concentrations are correlated with regional biomass of Atlantic cod in oceanic waters. Commun. Biol..

[33] Heupel, M. R. & Hueter, R. E. *Use of an Automated Acoustic Telemetry System to Passively Track Juvenile Blacktip Shark Movements BT - Electronic Tagging and Tracking in Marine Fisheries: Proceedings of the Symposium on Tagging and Tracking Marine Fish with Electronic Devices, Februar* (eds. Sibert, J. R. & Nielsen, J. L.) 217–236 (Springer, Netherlands, 2001).

[34] Castro JI (1996). Biology of the blacktip shark, *Carcharhinus limbatus*, off the southeastern United States. Bull. Mar. Sci..

[35] Compagno LJV, Dando M, Fowler SL (2005). A Field Guide to the Sharks of the World.

[36] Keeney DB, Heist EJ (2006). Worldwide phylogeography of the blacktip shark (*Carcharhinus limbatus*) inferred from mitochondrial DNA reveals isolation of western Atlantic populations coupled with recent Pacific dispersal. Mol. Ecol..

[37] Fields AT (2018). Species composition of the international shark fin trade assessed through a retail-market survey in Hong Kong. Conserv. Biol..

[38] Cardeñosa D, Quinlan J, Shea KH, Chapman DD (2018). Multiplex real-time PCR assay to detect illegal trade of CITES-listed shark species. Sci. Rep..

[39] Baremore IE, Passerotti MS (2013). Reproduction of the Blacktip Shark in the Gulf of Mexico. Mar. Coast. Fish..

[40] Killam KA, Parsons GR (1989). Age and growth of the blacktip shark, *Carcharhinus limbatus*, near Tampa Bay, Florida. Fish. Bull..

[41] Heupel MR, Simpfendorfer CA, Hueter RE (2004). Estimation of shark home ranges using passive monitoring techniques. Environ. Biol. Fish..

[42] Heupel MR, Hueter RE, Sibert JR, Nielsen JL (2001). Use of an automated acoustic telemetry system to passively track Juvenile Blacktip Shark movements bib. Electronic Tagging and Tracking in Marine Fisheries.

[43] Turner CR, Uy KL, Everhart RC (2015). Fish environmental DNA is more concentrated in aquatic sediments than surface water. Biol. Conserv..

[44] Knudsen SW (2019). Species-specific detection and quantification of environmental DNA from marine fishes in the Baltic Sea. J. Exp. Mar. Bio. Ecol..

[45] Edgar RC (2004). MUSCLE: Multiple sequence alignment with high accuracy and high throughput. Nucleic Acids Res..

[46] Klymus KE (2019). Reporting the limits of detection and quantification for environmental DNA assays. Environ. DNA.

[47] O’Donnell JL (2017). Spatial distribution of environmental DNA in a nearshore marine habitat. PeerJ.

[48] Yamamoto S (2017). Environmental DNA metabarcoding reveals local fish communities in a species-rich coastal sea. Sci. Rep..

[49] Jeunen GJ (2019). Environmental DNA (eDNA) metabarcoding reveals strong discrimination among diverse marine habitats connected by water movement. Mol. Ecol. Resour..

[50] Stat M (2019). Combined use of eDNA metabarcoding and video surveillance for the assessment of fish biodiversity. Conserv. Biol..

[51] Jeunen G (2019). Water stratification in the marine biome restricts vertical environmental DNA (eDNA) signal dispersal. Environ. DNA.

[52] Hunter ME (2017). Detection limits of quantitative and digital PCR assays and their influence in presence-absence surveys of environmental DNA. Mol. Ecol. Resour..

[53] Kralik P, Ricchi M (2017). A basic guide to real time PCR in microbial diagnostics: Definitions, parameters, and everything. Front. Microbiol..

[54] Hindson BJ (2011). High-throughput droplet digital PCR system for absolute quantitation of DNA copy number. Anal. Chem..

[55] Lehman RN (2019). An environmental DNA tool for monitoring the status of the Critically Endangered Smalltooth Sawfish, *Pristis pectinata*, in the Western Atlantic. Conserv. Genet. Resour..

