# Supplementary material for: Environmental DNA detection tracks established seasonal occurrence of blacktip sharks (Carcharhinus limbatus) in a semi-enclosed subtropical bay
Source: Sci Rep. 2020 Jul 16;10:11847. doi: 10.1038/s41598-020-68843-0 (PMC7367289; doi:10.1038/s41598-020-68843-0)
Supplement: Supplementary file 2 — Supplementary Information 2. [file 41598_2020_68843_MOESM2_ESM.docx]

**Manuscript title:** Environmental DNA tracks established seasonal occurrence of blacktip

sharks (*Carcharhinus limbatus*) in a semi-enclosed subtropical bay

**Authors**: Bautisse D Postaire^1*^, Judith Bakker ^1^, Jayne Gardiner ^2^, Tonya Wiley^3^_,_ Demian D

Chapman^1^

**Affiliations**: ^1^Department of Biological Sciences, Florida International University, 3000 NE

151st Street, North Miami, FL 33181, USA

^2^New College of Florida, Division of Natural Sciences, 5800 Bayshore Rd, Sarasota, FL 34243,

USA

^3^Havenworth Coastal Conservation, 5120 Beacon Road, Palmetto, Florida 34221, USA

**Corresponding author**: ^*^Correspondence to be addressed to: [postaireb@gmail.com](mailto:postaireb@gmail.com)

**Supplementary material 2**

Fishing gear details

**Gillnets**

A monofilament gillnet consisting of six different stretched-mesh size panels was used for sampling. Stretched-mesh sizes ranged from 7.6 cm to 14.0 cm in increments of 1.3 cm. Each panel was 3.0 m deep and 30.5 m long. The six panels were strung together and fished as a single gear (i.e., set). Sets were chosen randomly, and the gear was fished either perpendicular to shore or with the wind. All gillnet sets were made during daylight hours (07:00-18:00). For each set, mid-water temperature (degrees Celsius), salinity (PPT), and dissolved oxygen (milligrams per liter) were recorded using an environmental meter (YSI Inc.). Minimum and maximum depth was recorded from the vessel’s depth finder, and water clarity (depth of the photic zone) was measured with a secchi disc. Set soak time was defined from the time the gear started entering the water to the time the gear was removed completely from the water. Haul back typically started 0.5-1.0 hours after the gear first entered the water, while the net was checked directly when activity was observed prior to haul back.

**Longlines**

The bottom longline gear consisted of a 700 m braided nylon rope (8 mm) mainline, anchored and marked at either end with a large buoy, with up to 100 demersal gangions spaced at approximately 3-4 m intervals. Each gangion consisted of a stainless-steel tuna clip attached to 1m of 5mm braided nylon cord, a 6/0 stainless-steel swivel, 1 m of 1/16” stainless-steel wire, and a Mustad circle hook. A mixture of 12/0 - 16/0 hook sizes were used. Hooks were baited with a mixture of Spanish mackerel, ladyfish, bluefish, mullet, and Crevalle jack, to reflect the natural prey available in the bay. Longline soak times, calculated from the time the gear first entered the water until it was completely removed from the water, varied from 1 to 3 hours.

Sharks were identified, measured (pre-caudal length (PCL), fork length (FL), total length (TL), and stretch total length (STL)), to the closest half centimeter. Animals were sexed, assigned a life stage, and, if in good condition, tagged externally, and were subsequently released.
